# The critical role of phase difference in gamma oscillation within the temporoparietal network for binding visual working memory

**DOI:** 10.1038/srep32138

**Published:** 2016-08-30

**Authors:** Philip Tseng, Yu-Ting Chang, Chi-Fu Chang, Wei-Kuang Liang, Chi-Hung Juan

**Affiliations:** 1 Graduate Institute of Humanities in Medicine, Taipei Medical University, Taiwan; 2 Brain & Consciousness Research Center, Shuang-Ho Hospital, Taipei Medical University, Taiwan; 3 Institute of Cognitive Neuroscience, National Central University, Taiwan

## Abstract

How does the brain enable us to remember two or more object representations in visual working memory (VWM) without confusing them? This “gluing” process, or feature binding, refers to the ability to join certain features together while keeping them segregated from others. Recent neuroimaging research has reported higher BOLD response in the left temporal and parietal cortex during a binding-VWM task. However, less is known about how the two regions work in synchrony to support such process. In this study, we applied transcranial alternating current stimulation (tACS) over the left temporal and parietal cortex in gamma and theta frequency, with a phase difference of either 0° (in-phase) or 180° (anti-phase) to account for the different ways through which neural synchronization may occur. We found no facilitatory or inhibitory effect from sham, theta, and in-phase gamma stimulation. Importantly, there was an enhancement effect from anti-phase gamma tACS that was binding-specific, and such effect was only apparent in low-performing individuals who had room for improvement. Together, these results demonstrate that binding-VWM is supported by a temporally-precise oscillatory mechanism within the gamma frequency range, and that the advantageous 180°-apart phase relationship also implies a possible temporal driver-to-receiver time-lag between the temporal and parietal cortex.

How does the brain enable us to remember two or more object representations in visual working memory (VWM) without confusing them? This “gluing” process, or binding, refers to the ability to join certain features together while keeping them segregated from others in order to form correct object representations of the external world^1^,^2^. Neuroimaging investigations using feature-location binding paradigms (e.g., the classic color change detection paradigm) have mostly converged on the right parietal cortex, whose activities have been shown to be highly correlated or causal to one’s VWM capacity via the use of fMRI^3^, ERP^4^, EEG^5^, TMS^6^, and tDCS^7^,^8^. Notably, Shafritz, Gore and Marois^9^ observed higher BOLD response from the right parietal cortex when participants had to remember color-shape conjunctions simultaneously at different spatial locations, and not when the conjunctions were presented sequentially at the same location. This finding suggests that binding is mediated by the right parietal cortex when spatial location is one of the bound features, consistent with previous proposals of hemispheric asymmetry of spatial attention^10^.

Recently, to uncover the neural correlates of a purely featural-binding process that is not confounded by spatial locations, Parra, Sala, Logie, and Morcom^11^ had participants perform a color-shape binding VWM task where spatial locations of the stimuli were always different and randomized between displays, and thus irrelevant to the task. Interestingly, the right parietal cortex no longer shows any significant BOLD response. Instead, Parra *et al*. found higher BOLD responses from the *left* parietal cortex and left temporal cortex (fusiform gyrus) that were specifically responding to color-shape bindings over color-only or shape-only single features during the memory retention period. The authors speculated that the ventral temporal regions perhaps were responsible for processing multiple features, whereas the parietal cortex reflected the attention that was necessary to bind the features into objects. Regardless, these results suggest that VWM of feature bindings seems to rely on a left temporoparietal pathway that has not been previously implicated in the VWM literature. This opens up many important questions. For example, are the activities of the left temporal and parietal cortices merely correlational byproducts of other mediating regions, or are they causal to the binding processes? And if so, how do these regions coordinate their activities to mediate feature-binding?

One possible mechanism through which the left temporal and parietal cortex mediate the binding process is neural oscillation and synchronization, which have been shown to be important in cognitive functions like VWM that require fast and rapid neural assembly formations (and deformations) that cannot be done via long-term structural changes in the brain^12^. This transient nature of neural oscillation is especially of importance in binding, because different features such as color and shape are processed in different subregions of the brain, and thus need to be communicated in real-time and linked together to form one coherent object representation^13^. In particular, oscillations in the gamma frequency range (~40 Hz) may hold a special place in binding VWM due to its unique role in other perceptual binding phenomena such as the perception of coherent moving patterns^14^,^15^, coherent objects^16^,^17^, and color-location bindings^18^. For instance, Honkanen and colleagues^18^ observed stronger gamma oscillations when participants held color-location conjunctions in VWM, as opposed to separate, unbound features. As such, it is possible that the left temporal and parietal cortex from Parra *et al*.’s study^11^ coordinated their oscillatory activities in the gamma range to support nonspatial feature-binding in VWM. If so, this synchronization will also be dependent on the optimal phase difference between the two brain regions (0°~180°), where an in-phase (0°) or anti-phase (180°) synchronization-lag likely implies either an indirect or direct neural transmission, respectively (see Fell and Axmacher^19^, for a review).

The goal of this study is to explore the role of left temporoparietal-gamma synchronization in supporting binding in VWM. To do this, we combined the network of regions from the Parra *et al*.^11^ study in the context of binding VWM, with the literature on gamma oscillation in perceptual binding, to explore whether there may be a link between the two distinct bodies of literature. If positive, the current study would 1) establish a causal role for left temporal and parietal cortex in binding VWM, and 2) shed light on the oscillatory mechanism through which such temporoparietal network synchronizes its activities to support binding VWM. We utilized transcranial alternating current stimulation (tACS) to modulate the neural activities and synchronization of this network. tACS, in short, is a noninvasive electrical stimulation tool that applies alternating current in the form of sine waves over the scalp. The amplitude, frequency (delta~gamma), and phase lag (0°~180°) can all be fine-tuned in order to boost or attenuate the specific neural oscillation of interest^20^,^21^,^22^,^23^. With these unique strengths, tACS is ideally suited to test multi-regional network effects by modulating short- or long-range coherence between any two brain regions^24^,^25^. Here, in two experiments we tested the critical role of gamma oscillation (40 Hz) in binding VWM using either in-phase (Exp 1) or anti-phase (Exp 2) gamma tACS over the left temporoparietal network while controlling for the general tACS effect, if any, with identical task and procedures conducted in theta frequency.

## Experiment 1

**Figure 1:**
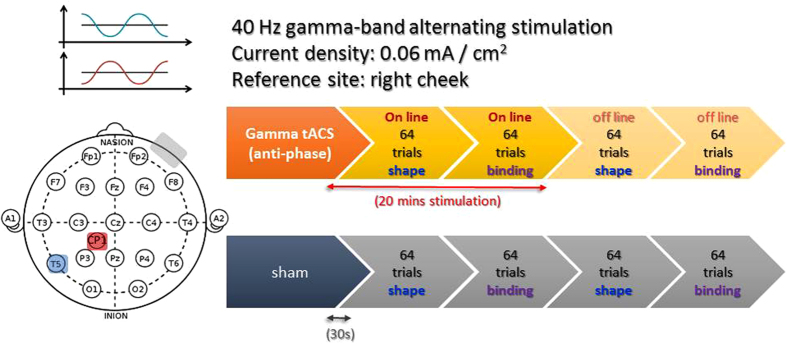
tACS protocol and experiment design for Experiment 1. tACS was applied via two electrodes (5 × 5 cm^2^) over the left temporal (T5) and parietal (CP1) sites, and a reference rubber electrode (5 × 7 cm^2^) over the right cheek. The stimulation waveform was sinusoidal at 40 Hz without DC offset at intensity of 1.5 mA (peak to peak), and the relative phase difference between the blue and red electrodes was set to 180°. Each participant performed on two different days (at least one week apart), one with sham (30 s) and one with active tACS (20 min), with counterbalanced order. On each day, the shape-only and shape-color binding trials were conducted in an interleaved block design.

In this experiment we tested the role of gamma synchronization within the left temporoparietal network with an anti-phase gamma tACS setup. Therefore, at 40 Hz gamma band, the left temporal and left parietal EEG signals are oscillating with 180° phase difference (Fig. 1, left panel). This anti-phase entrainment is often interpreted as disrupted synchronization between two given regions, which would lead to impaired cognitive performance^26^. However, since neural signals take time (at millisecond level) to be coded and transmitted between regions, a 0° phase difference with absolutely no time lag also seems implausible between any directly-connected regions^27^. Thus, an alternative account of the in-phase and anti-phase relationship is that an in-phase entrainment reflects an indirect connection, where the two regions are receiving a common input from a third mediating source, hence the 0° phase-lag (for a review, see Fell and Axmacher^19^). Anything beyond 0°, such as 180°, would then implicate a possible direct connection. In this light, the 180° anti-phase modulatory setup from the present experiment would not only provide causal evidence for the left temporoparietal network in VWM feature-binding, but also further differentiate the two theoretical views on in-phase and anti-phase coherence, where an impairment effect would support the de-synchronization view^26^,^28^ and an enhancement effect would support the direct-connection account^19^.

## Methods

### Participants

Twenty neurologically normal participants with normal or corrected-to-normal vision from National Central University, Taiwan, were recruited in this experiment (8 females, 12 males; mean age = 21). All participants gave written informed consent before participation. All experimental procedures were approved by the Institutional Review Board of the Linkou Chang Gung Memorial Hospital, Taoyuan County, Taiwan. All tasks, procedures, and stimulation protocols were carried in accordance to the IRB approval.

### Task and Procedures

Participants’ binding VWM performance was measured with a change detection task with two different conditions (shape-only vs. shape-color binding). The two conditions were conducted in a block design, with 2 dedicated blocks for the shape-only condition and 2 dedicated blocks for the feature-binding condition. The order of the blocks were always interleaved, resulting in two sequences that either started with a shape-only block or a binding block (see Fig. 1, right panel, for one example), which was counterbalanced across all participants. In addition, each participant came to the lab on two different days (at least one week apart), one with active tACS and one with sham tACS, and the order was also counterbalanced across participants. Within each participant, the order of the shape-only and binding blocks between the two days was kept the same. On both days, participants wore the tACS sponge electrodes throughout the entire experiments. Sham-tACS only lasted for 30 s, whereas active tACS as applied concurrently throughout the first two blocks, then switched off without participants’ notice as they moved on to the 3^rd^ and 4^th^ blocks. This design was done to keep active stimulation under 20 min for safety precautions and also comparability with the literature.

**Figure 2:**
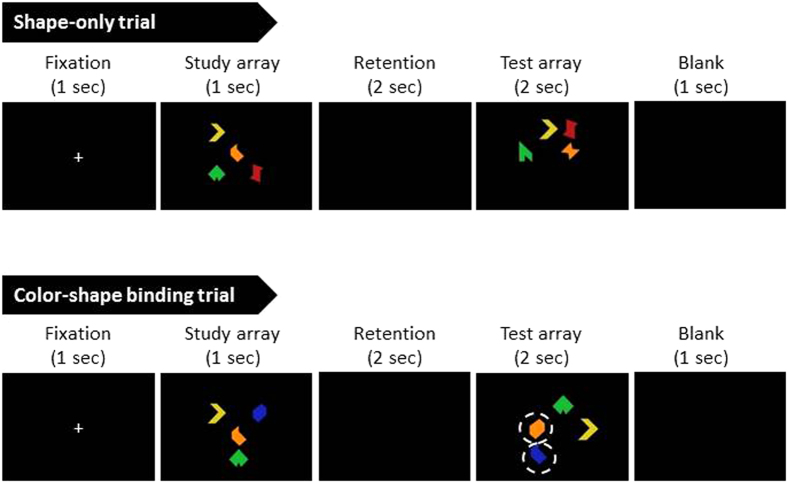
Examples of single-feature (shape) and feature-binding (color-shape) trials. All conditions consisted of a display of 4 items, where 2 items would change their appearance in a change trial. Locations would always change between the study and test array, in every item and every trial. Note that, although irrelevant, colors are added to shape-only trials as well in order to keep stimulus attributes identical between the shape-only and binding conditions. The white dotted circles (not visible to the participants) in the bottom panel denote the color-swapped shapes in a binding change trial.

Each block began with 12 practice trials, then 64 formal trials. In this task (Fig. 2), participants were first presented with a fixation cross (1 s), followed by the study array (1 s), retention interval (2 s), the test array (2 s), and ITI (1 s). Participants were instructed to remember the items presented in the study array, and judge whether they were same or different with the test array. The ratio between same- and change-trials was always 50:50, but this information was not disclosed to the participants.

In each trial, the set size was always 4, where 2 items would change their appearance on change trials. The stimuli were kept consistent with the Parra *et al*.^11^,^29^ studies, which consisted of a pool of 8 irregular-hexagonal shapes and 8 equiluminant colors (red, yellow, green, dark cyan, orange, blue, purple, gray). In change trials, in the shape-only condition, 2 shapes would remain the same while the other 2 would change to new shapes; in the shape-color binding condition, 2 colored shapes would remain the same while the other 2 shapes would swap color with each other such that none of the colors and shapes were new, but only their associations had changed. Like Parra *et al*.^11^, in order to avoid spatial locations as one confounding factor, we made locations irrelevant in the task by *always* changing the spatial locations between the study and test array, in every item, every trial. On every display, all items were presented within an 3 × 3 square grid on the screen where the size of each item is 3.6° × 3.6° in visual angle.

Note that a color-only condition was not included in the present experiment because our pilot data, as well as previous reports from other laboratory^11^, indicated a ceiling effect for color-only tasks, with accuracy approaching 1 even at set size of 4. Therefore, to better equate task difficulty between the single-feature and feature-binding conditions, the shape-only condition was chosen due to its higher level of difficulty. As another attempt to eliminate any difference between the shape-only and shape-color binding condition, we also presented the shape-only trials in colors. The colors would not change between the study and test array and thus cannot help participants perform better on the task, but provided an important stimulus control in terms of the physical and sensory attributes between the shape-only and shape-color binding conditions such that participants were seeing similar shape-color displays.

### Analysis

Participants’ hit and false alarm rates were used to compute the signal detection d′ index. The rationale of d′ in this study is a measurement of capability in discriminating whether a change has occurred. This index can be computed by Z (Hit rate) − Z (False alarm rate), which indicates the difference between the averages of the signal-present and the signal-absent distributions. By subtracting their z-scores, we can obtain a discrimination index that is not confounded from one’s tendency to respond yes or no.

### tACS Protocol

tACS was applied in this experiment through a constant current stimulation (DC-STIMULATOR MC NeuroConn) with three stimulation rubber electrodes: two electrodes (5 × 5 cm^2^) and a reference rubber electrode (5 × 7 cm^2^) covered by saline-soaked (NaCl solution) sponges. The two targeted regions, the superior parietal lobe (SPL) and the left temporal region, were identified based on the coordinates from the Parra *et al*.^11^ study in a separate group of 5 participants using Brainsight navigator system (Rogue Research Inc, Cardiff, UK). We then matched the EEG international 10–20 system^30^ to these two regions and used CP1 and T5 for the participants in the formal experiment. The reference electrode was then attached to the right cheek. The waveform of stimulation was sinusoidal at 40 Hz without DC offset at intensity of 1.5 mA (peak to peak). The relative phase difference between two brain regions was set to 180°. As previously mentioned, participants wore the tACS sponge electrodes throughout the entire experiments on both sham and active tACS days. Active tACS was applied during the first 2 blocks for 20 m, which is enough to elicit an after-effect of 40 m or longer^31^,^32^, and sham-tACS only for 30 s. On both days, none of the participants reported any noticeable skin sensations or perception of phosphene.

## Result

Participants’ d′ scores were analyzed with a repeated measures 2 × 2 × 2 ANOVA with factors of task-condition (shape only vs. binding), stimulation (sham vs. active), and session (online: session 1 & 2; offline: session 3 & 4). There was a significant main effect of task condition [F(1, 19) = 50.268, p < 0.001; ɳ^2^ = 0.726], presumably because binding was more challenging than shape-only changes. There was no significant main effect of stimulation [F(1, 19) = 2.165, p = 0.158; ɳ^2^ = 0.102], but a main effect of session was marginally significant [F(1, 19) = 3.931, p = 0.062; ɳ^2^ = 0.171], implying the presence of a slight practice effect. Importantly, there was a significant interaction between task condition and stimulation [F(1, 19) = 6.67, p = 0.018; ɳ^2^ = 0.26] while all other two-way and three-way interactions were not significant (task-condition and session: [F(1, 19) = 0.021, p = 0.888; ɳ^2^ = 0.001]; stimulation and session: [F(1, 19) = 0.508, p = 0.485; ɳ^2^ = 0.026]; three-way interaction: [F(1, 19) = 1.198, p = 0.287; ɳ^2^ = 0.059]).

To make sure that the interaction between task condition and tACS would remain significant when the factor of session is not in the mix, we again conducted a repeated measures 2 × 2 ANOVA on each participant’s merged overall d′ score across all sessions. Both the main effect of task condition [F(1, 19) = 43.301, p < 0.001; ɳ^2^ = 0.695] and the interaction between task condition and tACS [F(1, 19) = 6.324, p = 0.021; ɳ^2^ = 0.25] remained statistically significant, while the main effect of tACS remained non-significant [F(1, 19) = 2.26, p = 0.149; ɳ^2^ = 0.106]. To explore this persistent interaction between task condition and tACS, separate comparisons showed that tACS application only had an effect on participants’ binding VWM performance [sham d′: 1.39; tACS d′: 1.69; t_(19)_ = 2.969, p = 0.014], and the same effect was absent in the shape-only condition [sham d′: 2.00; tACS d′: 2.00; t_(19)_ = 0.002, p = 0.999]. Here it is important to note that the effect of anti-phase tACS actually enhanced participants’ VWM performance, which is more in line with the direct-connection account raised by Fell and Axmacher^19^.

**Figure 3:**
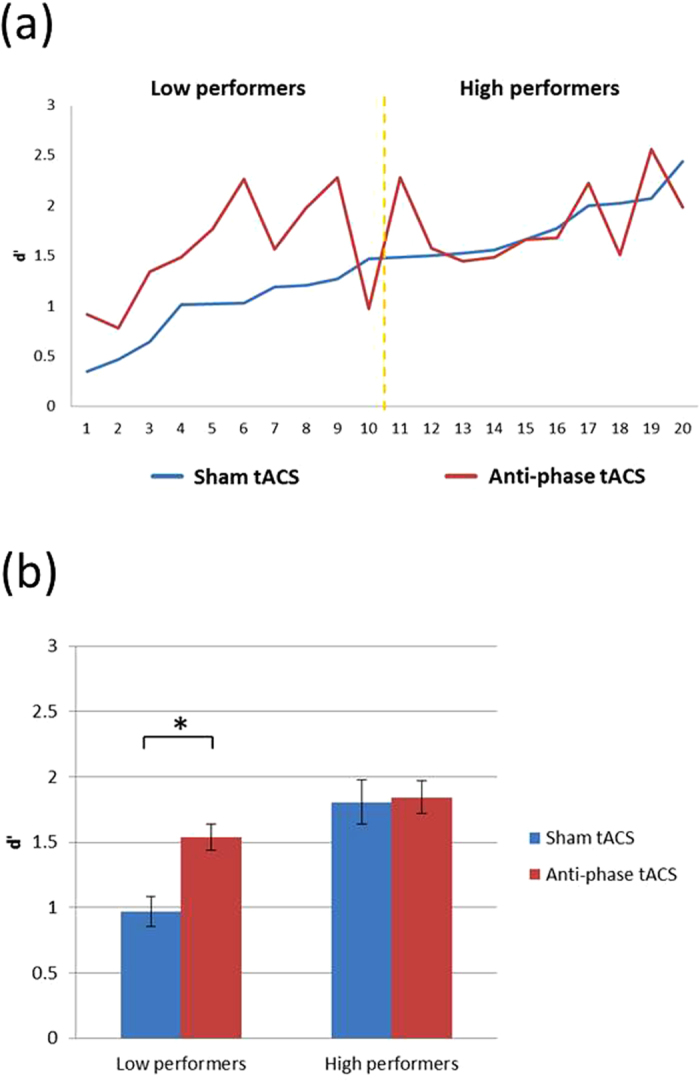
(**a**) Individual Differences in Experiment 1. Effect of anti-phase 40 Hz tACS interacted with preexisting individual differences in binding-VWM performance. The X axis represents each individual participant, and the Y axis represents their binding memory performance from the sham (blue) and tACS (red) condition. The yellow dotted line represents the cutoff line for the median split, with the low-performers on the left and the high-performers on the right. (**b**) Results of Experiment 1. Anti-phase 40 Hz tACS was only facilitative in the low-performing group in binding VWM, and not the high-performing group. The error bars represent standard errors.

Since we have observed an enhancement effect from tACS, we lined up the participants based on their sham d′ from lowest to highest and examined each individual’s tACS effect (Fig. 3a). This was done based on our previous findings on the interaction between tDCS and individual differences in VWM enhancement^7^,^8^, where low-performers seemed to benefit the most from the stimulation. From Fig. 3a it is clear that these 20 participants varied widely in their binding VWM (d′ range: 0.35~2.44), much like our 2012 findings. Therefore, based on each participant’s performance in the binding sham condition (blue line in Fig. 3a), we divided the participants via a median split into a low-performer group (n = 10; mean d′ = 0.968) and a high-performer group (n = 10; mean d′ = 1.805) and conducted a separate 2 × 2 ANOVA with the factors of tACS (sham vs. active) and performer baseline (low vs. high). There was a significant main effect of stimulation [F(1, 18) = 9.776, p = 0.006; ɳ^2^ = 0.352] and performer baseline [F(1, 18) = 13.115, p = 0.002; η^2^ = 0.422]. Critically, there was a significant interaction between the two factors [F(1, 18) = 7.556, p = 0.013; ɳ^2^ = 0.296]. Separate comparisons revealed that tACS was only facilitative in the low-performing group [t_(9)_ = 3.846, p = 0.004], but not in the high-performing group [t_(9)_ = 0.293, p = 0.776] (Fig. 3b). In addition, there was no significant difference between online and offline sessions in these low- [t_(9)_ = −0.683, p = 0.512] and high-performers [t_(9)_ = −0.584, p = 0.574], suggesting that the effect of tACS at least lasted throughout the offline session for the improved low-performers.

Lastly, to test whether there was a criterion shift induced by active tACS in addition to increased signal-to-noise ratio, we analyzed these low-performers’ hit rate and false alarm rate separately to see if one was more heavily influenced than the other. Both numbers were statistically significant, where tACS increased the hit rate by 7% [t(9) = 3.336, p = 0.009] and decreased the false alarm rate also by 7% [t(9) = 2.415, p = 0.039]. We also used C as an index for response bias [C = −0.5*(Z(Hit)+Z(FA))], where a 0 represents bias-free response and a positive value denotes conservative criterion (i.e., more likely to say no change). We observed a C of 0.28 for the sham condition and C of 0.38 for the active tACS condition [t(9) = 2.01, p = 0.075], suggesting a marginally significant shift towards conservative responses in participants after active tACS, in addition to their improved binding performance.

## Discussion

The present study aimed to examine whether left temporoparietal regional activities are causal to one’s binding VWM performance. To do this, we applied anti-phase 40 Hz gamma tACS to entrain the phases between left temporal and parietal cortices to be 180° apart. By doing so we also tested the two competing accounts, the synchronization account^26^,^28^ and the direct-connection account^19^, that have been proposed to explain the significance of interregional phase differences. In short, we observed an improvement, instead of impairment, effect from anti-phase 40 Hz tACS in low-performers’ binding VWM performance. This improved performance was not only observed in the online session, but also lasted throughout the 20-min offline session, suggesting an after effect duration of at least 20 min for our tACS protocol. Together, these observations have several implications for the neural mechanisms behind both binding VWM and tACS: 1) the left temporal and parietal cortices are causally involved in binding VWM, 2) 40 Hz gamma oscillation is also causally involved in this temporoparietal pathway in supporting binding VWM, 3) enhanced memory performance from anti-phase entrainment supports the direct-connection account, which possibly implies a direct sender-and-receiver relationship between left temporal and parietal cortices. We discuss each of these three points below.

First, with the aid of brain stimulation, we can now conclude that both temporal and parietal cortices are critically involved in binding VWM. It is important to note that we only observed a modulation effect in the binding condition and not the shape-only condition. Considering that the physical attributes of the stimuli between the shape-only and binding conditions are virtually identical, and that both of these conditions require the use of VWM, the only major difference between these two conditions is whether binding is needed. Therefore, it is plausible to conclude that the left temporoparietal pathway is binding-specific, and functionally different from the right posterior parietal cortex that is frequently implicated in the single-feature VWM literature. To be cautious though, it is also possible that the shape-only performance was too high (mean d′ = 2) for any tACS effect to show through, therefore the lack of any tACS modulatory effect may be due to a ceiling effect. However, given the converging evidence from previous fMRI study^11^, and the fact that splitting our participants into low- and high-performers based on shape-only performances still did not yield any significant tACS effect in either groups, we think the likelihood of a ceiling effect here is low.

Second, our tACS results demonstrated the importance of 40 Hz gamma oscillation in improving binding VWM. There have been studies suggesting a role for gamma oscillation in perceptual grouping^16^ and single-feature working memory^33^,^34^. The present study links the two together and demonstrates a causal role for gamma oscillation in maintaining bound visual representations in visuospatial working memory. Note that this does not necessarily exclude other frequency bands from binding VWM. For example, theta-locked gamma oscillation is often reported in the general working memory literature^35^,^36^, albeit less in the visual domain. Therefore, it is possible that increased gamma oscillation has produced a rippling effect in other coupled frequencies to support binding VWM. This question will require the use of simultaneous EEG to answer. Nevertheless, our results on 40 Hz gamma suggest that gamma frequency is a critical mechanism to the cognitive processes behind binding VWM, and is not a mere harmonic artifact of other lower frequencies as frequently reported by EEG studies that apply linear transforms such as Fourier or Wavelet analysis to nonlinear EEG data (for a review, see Huang and Wu^37^).

Lastly, and perhaps the more surprising aspect of our findings, is the improvement effect from 180° anti-phase gamma tACS. This is in contrast with previous tACS studies that have dichotomized in-phase and anti-phase signals as representations of coherent and incoherent neural communication, respectively^26^,^28^, where anti-phase stimulation usually disrupts interregional coherence. Here it is important to note that, regardless of 0° or 180° phase differences, two signals can achieve perfect coherence in both cases as long as the precise phase difference, be it 0° or 180°, is kept consistent over time^38^. In this light, the 40 Hz stimulation applied here actually has high coherence, with a consistent 180° phase difference between the left temporal and parietal cortex. This anti-phase improvement finding is also in line with electrophysiological data from awake-behaving monkeys that show a 152° phase difference (roughly corresponds to 8~13 ms lag) between the frontal and occipital cortex while performing a visual detection task^27^. Thus, our results here are consistent with the idea proposed by Fell and Axmache^19^, which suggests that regions with direct transmission are bound to have certain phase lag above 0°.

## Experiment 2

**Figure 4:**
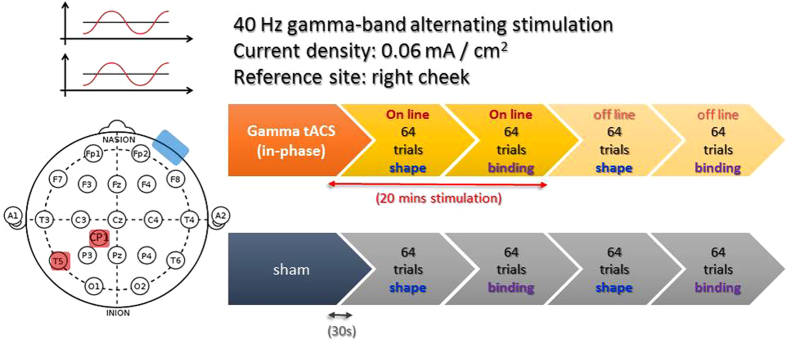
tACS protocol and experiment design for Experiment 2. tACS was applied via two electrodes (5 × 5 cm^2^) over the left temporal (T5) and parietal (CP1) sites, and a reference rubber electrode (5 × 7 cm^2^) over the right cheek. The stimulation waveform was sinusoidal at 40 Hz without DC offset at intensity of 1.5 mA (peak to peak), and the relative phase difference between the two electrodes (red) was set to 0°. Each participant performed on two different days (at least one week apart), one with sham (30 s) and one with active tACS (20 min), with counterbalanced order. On each day, the shape-only and shape-color binding trials were conducted in an interleaved block design.

In Experiment 1 we observed improved binding VWM performance in low-performers when anti-phase gamma tACS was applied over the left temporal and parietal cortex. Based on this finding, it is important to investigate whether phase difference within the temporoparietal network is crucial to binding VWM at all. In other words, it is possible that 0° in-phase stimulation can also produce the same pattern of results if 1) the left temporal and parietal cortices can communicate with each other via gamma oscillation that is unspecific to phase differences, and 2) the results of Experiment 1 was due to a general effect of electricity travelling through the cortex. To test these possibilities, in Experiment 2 we employed an in-phase tACS setup with the exact same task, design, stimulation regions and frequency. Thus, at 40 Hz gamma frequency, the left temporal and left parietal signals are oscillating with 0° phase difference (Fig. 4). If the out-phase gamma coherence setup from Experiment 1 was crucial to binding-VWM, then in-phase tACS with the same frequency over the same regions should either lead to an impairment or have no effect on VWM performance.

## Methods

### Participants

Twenty neurologically normal participants from National Central University who did not participate in Experiment 1 took part in the present experiment (8 females, 12 males; mean age = 23). All participants had normal or corrected-to-normal vision. All participants gave written informed consent prior to their participation. All experimental procedures were approved by the Institutional Review Board of the Linkou Chang Gung Memorial Hospital, Taoyuan City, Taiwan. All tasks, procedures, and stimulation protocols were carried in accordance to the IRB approval.

### Task, Procedures, and tACS Protocols

All tasks and procedures were identical as Experiment 1, with the exception of in-phase tACS protocol. Specifically, the phase difference between the two electrodes in this experiment is set to 0° to achieve maximal synchronization (Fig. 4).

## Results

Participants’ d′ scores were submitted to a repeated-measures 2 × 2 × 2 ANOVA with factors of task-condition (shape only vs binding), stimulation (sham vs. active) and session (online: session 1 & 2; offline: session 3 & 4). There was a significant main effect of task-condition [F(1, 19) = 11.525, p < 0.003; ɳ^2^ = 0.378] and session [F(1, 19) = 9.494, p = 0.006; ɳ^2^ = 0.333], with no main effect for stimulation [F(1, 19) = 0.633, p = 0.436; ɳ^2^ = 0.032]. These main effects are consistent with the observations from Experiment 1, namely that binding is more difficult than shape-only change detection, and that participants improved their overall performance as time went on. Importantly, unlike Experiment 1, there was no significant interaction between task-condition and stimulation [F(1, 19) = 1.499, p = 0.236; ɳ^2^ = 0.073]. All other two-way and three-way interactions were not significant either (task-condition and session: [F(1, 19) = 0.301, p = 0.589; ɳ^2^ = 0.016]; stimulation and session: [F(1, 19) = 0.018, p = 0.896; ɳ^2^ = 0.001]; three-way interaction: [F(1, 19) = 0.695, p = 0.415; ɳ^2^ = 0.035]). Merging d′s together across sessions yielded similar results in a new repeated measures 2 × 2 ANOVA: a significant main effect of task-condition [F(1, 19) = 10.825, p = 0.004; ɳ^2^ = 0.363], but no effect of stimulation [F(1, 19) = 0.428, p = 0.521; ɳ^2^ = 0.022] or the interaction between task-condition and stimulation [F(1, 19) = 1.234, p = 0.28; ɳ^2^ = 0.061]. Separate comparisons like we did in Experiment 1 also did not reveal any effect in either the shape-only condition [t_(19)_ = 0.910, p = 0.374] or the feature-binding condition [t_(19)_ = 0.158, p = 0.876]. Therefore, in-phase gamma tACS had no effect in either direction on shape-only and binding VWM.

**Figure 5:**
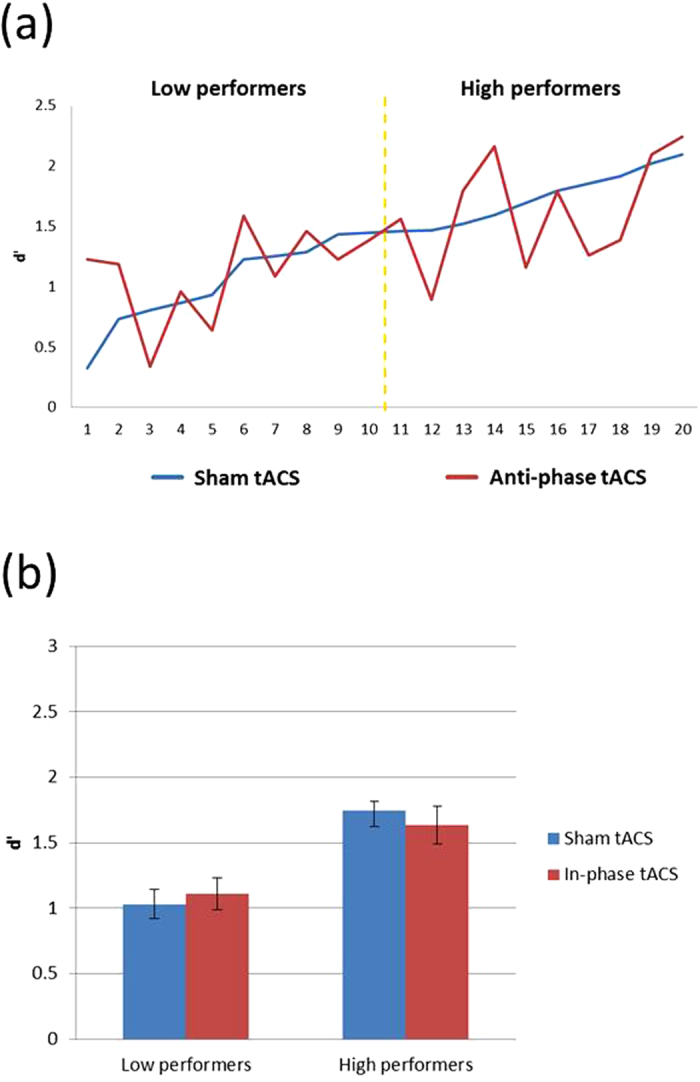
(**a**) Individual Differences in Experiment 2. Like Experiment 1, participants also varied widely in terms of their binding-VWM performance. However, there was no interaction between in-phase 40 Hz tACS and such individual differences in binding-VWM performance. The X axis represents each individual participant, and the Y axis represents their memory performance from the sham (blue) and tACS (red) condition. The yellow dotted line represents the cutoff line for the median split based on binding sham performance, with the low-performers on the left and the high-performers on the right. (**b**) Results of Experiment 2. In-phase 40 Hz tACS did not produce behavioral modulation effect in either low- or high-performers.

Despite the absence of a binding-specific effect from tACS, it remains possible that such effect may emerge when we take participants’ VWM capacity into account. We again split the participants into low- (n = 10; mean d′ = 1.03) and high-performers (n = 10; mean d′ = 1.74) based on their sham d′, which are comparable with those from Experiment 1 (0.968 and 1.805 for low and high, respectively) (Fig. 5a). However, unlike Experiment 1, the interaction term between tACS (active vs. sham) and performer (low vs. high) in the repeated measures 2 × 2 ANOVA was not significant [F(1,18) = 1.026, p = 0.325; ɳ^2^ = 0.054]. Separate comparisons of the effect of tACS on binding VWM were also not significant in both low- [t_(9)_ = 0.611, p = 0.556] and high-performers [t_(9)_ = 0.819, p = 0.434] (Fig. 5b).

## Discussion

The present experiment was designed to clarify whether feature-bindings in VWM require a specific phase difference within gamma oscillations between the left temporal and parietal cortex. To this end, we applied in-phase tACS over the same brain regions and using the same frequency as Experiment 1 to provide a good comparison. In short, we did not observe any modulation effect with in-phase tACS, not in the shape-only condition or the binding condition. Even if we follow through the analysis procedure from Experiment 1 and split the participants based on their performance baseline, there was still no evidence of any behavioral modulation in either group. Therefore, by simply changing the phase parameter to 0°, we have eliminated the improvement effect of gamma tACS in low-performers despite everything being consistent with Experiment 1.

The first implication of these results here is that the improvement effect we have observed from Experiment 1 was not a mere byproduct of electricity flowing through the brain. This is important because sham conditions, although procedurally identical to the active session, sometimes cannot control for the effects of brain stimulation as adequately as frequency-controlled or region-controlled sessions. Therefore, to a certain extent the present experiment serves as a better comparison against Experiment 1 due to its identical stimulation protocol except the phase difference 0°.

Second, the absence of any tACS modulation effect here also suggests that binding VWM is supported by a temporally-precise oscillatory mechanism between the left temporal and parietal cortex. According to Fell and Axmacher^19^, a phase lag beyond 0° between two regions is more in line with the idea of direct communication. This is consistent with our results from both experiments, and is supported by the fact that our in-phase tACS did not produce a facilitation effect like other studies did^26^. However, one cautious note regarding this interpretation is that, strictly speaking, Fell and Axmacher’s account would predict an impairment effect with in-phase tACS because forcing two neural populations to oscillate with suboptimal phase differences is likely to generate noise and interference, which we did not observe. This lack of impairment effect also cannot be attributed to a floor effect in our participants because even our high-performers did not show any tACS effect in either direction. Therefore, to what extent are in-phase and anti-phase signals complete opposites of each other, with the consequence of complete opposite behavioral outcomes, remains to be explored (see Struber *et al*.^28^ for another example of positive effect with anti-phase tACS and null-effect with in-phase tACS). Nevertheless, our results here clearly indicate the importance and precise nature of gamma oscillation and phase differences within the left temporoparietal network in supporting feature-binding in VWM.

## Control Experiments

In Experiment 1 and 2, we applied anti-phase and in-phase 40 Hz tACS over the left temporoparietal network and observed improved binding VWM performance versus no effect, respectively. To ensure and test whether the observed effect was specific to anti-phase gamma frequency versus a general anti-phase effect across the entire frequency spectrum (which would be an interesting and informative finding on its own), the present control experiment was designed to mimic Experiment 1 and 2 using in-phase and anti-phase theta (6 Hz) tACS. Theta band was chosen here because in-phase theta tACS over the left frontoparietal network has been demonstrated to be facilitative to color VWM change detection^39^, making it a suitable choice due to its relevance in VWM.

## Methods

### Participants

Twenty neurologically normal participants from National Central University who did not participate in Experiment 1 and 2 took part in the in-phase theta experiment (8 females, 12 males; mean age = 22). A separate group of twenty (8 females, 12 males; mean age = 22) who also did not participate in any of the previous experiments participated in the anti-phase theta experiment. All forty participants had normal or corrected-to-normal vision. All participants gave written informed consent prior to their participation. All experimental procedures were approved by the Institutional Review Board of the Linkou Chang Gung Memorial Hospital, Taoyuan City, Taiwan. All tasks, procedures, and stimulation protocols were carried in accordance to the IRB approval.

### Task, Procedures, and tACS Protocols

All tasks and procedures were identical as Experiment 1 and 2, including in-phase and anti-phase manipulations, with the only exception of tACS frequency. That is, the stimulation frequency in the present experiment was set to 6 Hz.

## Results and Discussion

In both experiment, participants’ d′ scores were submitted to a repeated-measures 2 × 2 × 2 ANOVA with factors of task-condition (shape only vs binding), stimulation (sham vs. active), and session (online vs. offline). In the in-phase theta experiment, there was a significant main effect of task-condition [F(1, 19) = 12.058, p = 0.003; ɳ^2^ = 0.388], a marginally significant effect of session [F(1, 19) = 3.957, p = 0.061; ɳ^2^ = 0.172], and no effect for stimulation [F(1, 19) = 0.067, p = 0.798; ɳ^2^ = 0.004], all of which are similar to our observations from Experiment 1 and 2. Critically, there was no significant interaction between task-condition and stimulation [F(1, 19) = 0.044, p = 0.835; ɳ^2^ = 0.002], and all other two-way and three-way interactions were not significant (task-condition and session: [F(1, 19) = 2.284, p = 0.147; ɳ^2^ = 0.107]; stimulation and session: [F(1, 19) = 1.033, p = 0.322; ɳ^2^ = 0.052]; three-way interaction: [F(1, 19) = 0.148, p = 0.704; ɳ^2^ = 0.008]). Like Experiment 1 and 2, we merged participants’ d′ scores across sessions and re-conducted a repeated measures 2 × 2 ANOVA between task condition and stimulation. There was a significant main effect of task-condition [F(1, 19) = 12.252, p = 0.002], but no effect of stimulation [F(1, 19) = 0.02, p = 0.89] or the interaction between task-condition and stimulation [F(1, 19) = 0.004, p = 0.95].

In the anti-phase experiment, our 2 × 2 × 2 ANOVA showed a significant main effect of task-condition [F(1, 19) = 13.662, p = 0.002; ɳ^2^ = 0.418], with no main effect for stimulation [F(1, 19) = 1.783, p = 0.198; ɳ^2^ = 0.086] and session [F(1, 19) = 0.289, p = 0.597; ɳ^2^ = 0.015]. There was no significant interaction between task-condition and stimulation [F(1, 19) = 0.453, p = 0.509; ɳ^2^ = 0.023], and all other two-way and three-way interactions were not significant as well (task-condition and session: [F(1, 19) = 0.012, p = 0.913; ɳ^2^ = 0.001]; stimulation and session: [F(1, 19) = 0.007, p = 0.934; ɳ^2^ < 0.001]; three-way interaction: [F(1, 19) = 1.508, p = 0.234; ɳ^2^ = 0.074]). Merging d′ scores together across sessions yielded similar results: a significant main effect of task-condition [F(1, 19) = 12.349, p = 0.002], but no effect of stimulation [F(1, 19) = 1.602, p = 0.221] and no interaction between task-condition and stimulation [F(1, 19) = 0.437, p = 0.517].

Together, the present control experiments using in-phase and anti-phase theta protocol found no causal evidence of left temporoparietal theta tACS in improving or impairing feature-binding VWM. These results suggest that the improvement effect from Experiment 1 was specific to anti-phase gamma oscillation, and was not due to a general byproduct of anti-phase tACS.

## General Discussion

The goal of the present study was twofold: 1) to test the causal role for the left temporal and parietal cortex in binding VWM, and 2) to shed light on the oscillatory mechanism on which such temporoparietal network operates. To this end, 40 Hz sinusoidal stimulation was applied via tACS over these two regions in both anti-phase (Experiment 1) and in-phase (Experiment 2) fashion. We observed improved VWM for bound representations only in low-performers, and only in the anti-phase tACS condition (Experiment 1) – where the two regions were oscillating with 180° phase difference in time. No improvement or impairment effect was observed in the in-phase tACS condition, in both low- and high-performers (Experiment 2), as well as the two control experiments that used identical setups and procedures except in-phase and anti-phase theta tACS. Moreover, the positive effect from Experiment 1 was specific to the color-shape binding condition, and was absent in the shape-only single-feature condition. This is consistent with the reports from Parra *et al*.^11^, whose fMRI results showed increased BOLD response in the left temporal and parietal cortex when maintaining bound representations in VWM. The findings from the current study establish a causal role for this temporopraietal network, and further suggest that it relies heavily on temporally-shifted (i.e., 180°) gamma-frequency oscillations to possibly code and maintain bound color-shape items^40^. We conclude that memory of bound visual representations is dependent upon a temporally-precise oscillatory mechanism that involves gamma coherence between the left temporal and parietal cortex.

### Gamma and other frequency oscillations in VWM

The importance of gamma oscillation in cognitive functioning has received much empirical support lately, especially now that neural oscillation is viewed as a mechanism for interregional communication due to its unique frequency- and timing-specific nature^41^. In the case of gamma oscillation, it has been shown to mediate the propagation of information during both bottom-up and top-down processing^12^. Consequently, changes in gamma oscillation have been observed in multiple cognitive domains such as attention^42^, perception^43^, and working memory^40^. Even under working memory, gamma amplitude has been shown to be correlated with the number of items that need to be maintained^44^,^45^,^46^, and is also implicated in working memories of multiple modalities such as visual^33^,^44^,^47^, auditory^48^,^49^,^50^,^51^, and somatosensory^52^ working memory. Combining these findings with Tallon-Baudry’s report^16^ of increased gamma synchronization when participants successfully “bind” the three corners of the Kaniza’s triangle together to perceive an illusory surface (also see Rose and Buchel^53^), it is reasonable that our study has found a critical role for gamma synchronization in maintaining feature-binding representations in VWM. However, it is important to note that the effect of anti-phase gamma tACS was very specific to binding memory and did not benefit non-spatial, single-featured, shape-only representations. Thus, it is possible that gamma oscillation may be more specific than previously implicated, and may not be involved in all kinds of working memory processing. Another more plausible view is that perhaps gamma is involved in multiple kinds of memory processing, but works differently depending on the brain regions and different temporal phase shifts ranging between 0° and 180° depending on the context. Therefore, non-spatial and non-binding representations may also rely on gamma oscillatory activities, but perhaps differ from the current study in terms of their phase-shift, participating brain regions, specific gamma frequency range (i.e., 30~80 Hz), as well as other participating low-frequencies in the form of cross-frequency couplings^35^,^40^,^54^,^55^.

The absence of a causal effect of theta tACS on binding VWM may be surprising for some since theta tACS has been demonstrated to be able to improve color VWM performance by Jausovec and Jausovec^39^. Therefore, in the control experiments one might have predicted a significant interaction between task type and tACS in favor of shape change detection, or a main effect of tACS that boosts performance in both shape-only and binding trials (since successful shape VWM should also indirectly benefit shape-color binding performance). However, it is important to note two critical differences between our and the Jausovec and Jausovec study: task design and brain regions. First, regarding task design, the present binding paradigm was modeled after Parra and colleagues’ 2010 and 2014 studies^11^,^29^, where spatial locations were always randomized and thus irrelevant to the binding processes. This allowed our task to focus on feature-binding between color and shape, and is quite different from a standardized change detection task that our previous studies, as well as Jausovec and Jausovec, have used^6^,^7^,^8^,^39^, where spatial locations between the study and test array always remain stable. Therefore, the change detection task in essence is a color-location binding task. Second, and perhaps more important, is the difference between stimulated regions. Where previous study by Jausovec and Jausovec applied theta tACS over the left frontal and parietal cortex, in this control experiment we applied theta tACS over the left temporal and parietal cortex. The fact that the effect of tACS can disappear simply by changing one of the brain regions highlights the importance of stimulation site(s) even when stimulation protocols are held constant. In other words, the present control experiment can be viewed as a regional-control experiment of the Jausovec and Jausovec study; and the results from both studies together demonstrate that tACS effects are quite regionally specific. This should also hopefully ameliorate the concern that some may have towards tACS’ regional specificity current spreading; if that were the case, we should have observed similar effects as Jausovec and Jausovec by stimulating different brain region(s). We revisit this point of focality again in the next section.

If spatial locations and brain regions are not adequate to explain the lack of theta effect, it is possible that our anti-phase gamma tACS had induced changes in multiple oscillating frequencies that are coupled with gamma band, including theta. This speculation is based on the observation that cross-frequency couplings have often been reported, especially theta-gamma^40^,^41^ and alpha-gamma^42^ couplings in various forms such as phase-amplitude or phase-phase modulation. Although the precise role for each frequency is still under rigorous investigation, many have proposed a memory-related function for theta and gamma frequency, and inhibition of task-irrelevant information for alpha frequency. More specifically, some have proposed that theta and gamma frequencies are responsible for the organization and maintenance, respectively, of working memory representations, hence the close coupling^40^. Others have suggested a group-item relationship between theta and gamma such that the limit of working memory capacity stems from the limited number of gamma cycles enclosed within a theta cycle^35^,^54^. Importantly, Pahor and Jausovec found that theta tACS not only influenced theta oscillation, but changed alpha power throughout different regions as well. Therefore, we entertain the possibility that our anti-phase gamma tACS from Experiment 1 has also induced oscillatory fluctuations in other frequencies that worked in conjunction with enhanced gamma to mediate binding VWM.

### Interregional distance as an important modulator of tACS effects on phase relationship

By applying gamma tACS of different phase relationships over the left temporal and parietal cortex, the present study was able to test the predictions of two competing accounts, the synchronization account^26^ and the direct-connection account^19^, that have been proposed to explain the significance of interregional phase differences. Our results are in sharp contrast with previous tACS studies that have observed a facilitation effect with in-phase stimulation^26^,^31^,^32^. Upon close examination of each of these studies, we think the interaction between interregional distance and tACS frequency band may be a determining factor that can reconcile the inconsistencies, which we describe below.

Polanía and colleagues^26^ were the first to utilize tACS with in-phase and anti-phase modulations to influence cognitive functioning. These authors applied theta (6 Hz) tACS (0° or 180° phase difference) over the left frontal and parietal cortex, and measured cognitive performance via a letter working memory task. They found faster and slower reaction times to be associated with in-phase and anti-phase theta stimulation, respectively. Most important, control experiment with gamma (35 Hz) tACS did not generate any results. Similarly, Jausovec and colleagues have also done a series of tACS studies applying in-phase theta stimulation over the left frontal or parietal cortex, with the reference electrode located over the right eyebrow, and reported improved performance on visuospatial memory recall^31^, recognition^39^, and Raven’s matrices^32^. Note that in these studies done by Jausovec and colleagues, no anti-phase comparison condition was available, and that the reference electrode was always placed over the right eyebrow, a point of particular interest that we revisit in the next section.

The common ground between these studies above is that they all used theta stimulation (~6 Hz) over long-distance connections (left frontal to left parietal, left frontal to right forehead, left parietal to right forehead, or right parietal to right forehead). These setups are quite different from the current study, where gamma or theta tACS was applied over a relatively shorter distance (left temporal to left parietal). One compelling explanation for the discrepancies is that theta band is more suitable for long-range transmissions between distal regions due to its longer wavelength characteristics, whereas gamma is more suitable for proximal regions for the same reason^56^. Indeed, one recent tACS study done by Strüber *et al*.^28^ that found effective anti-phase gamma effect in shaping one’s bistable perception also had a similar short-range electrode placement (left parietal to left occipital, right parietal to right occipital). Importantly, such short-range arrangement yielded no tACS effect when stimulation frequency was switched to theta band, just like what we have observed. Therefore, although it is still unclear how distance between the two electrodes may interact with stimulation frequency, data from most tACS studies as well as the current study so far do agree on the long and short range properties of theta and gamma oscillation, respectively.

On a related note, the extent of focality of tDCS and tACS should also be considered cautiously when interpreting results like the current study. This is because most of the current does not go beyond the skin^57^, and even when it does, current inside the skull can be diffused by CSF^58^. For tDCS, although highest current density does occur beneath the target and reference electrodes, regions that are adjacent^59^, functionally connected^60^, signally complex^61^, or in between the electrodes^62^ can also show higher current densities or activation. Therefore, the focality or the current spread of tACS is dependent upon the location and distance between the electrodes^63^,^64^,^65^.

While there may be multiple other contributing factors beyond the interaction between interregional distance, current spread, and stimulation frequency, one clear message from the current study is that in-phase and anti-phase coherence should not be taken as pure evidence of synchronization and desynchronization that are completely opposite of each other with opposite effects on cognitive functioning, at least not in gamma band. Indeed, if this was true, we should have observed an impairment effect in Experiment 2 (in-phase condition), which was not the case either in our high-performers or the entire group as a whole. Therefore, a wider network perspective that incorporates brain regions, distance, phase shifts, task, and phase-phase or phase-amplitude coupling is suggested when considering results from in- and anti-phase tACS. Importantly, the Strüber *et al*. study^28^ that found increased vertical bistable perception with anti-phase gamma tACS also did not report increased horizontal perception with an in-phase gamma setup. Therefore, when looking at brain stimulation studies that manipulate in- and anti-phase coherence among different regions, it is suggested that one should consider a wider network perspective that incorporates interregional distance and phase shifts, as well as other possible factors such as the cognitive task at hand, phase-phase or phase-amplitude coupling with other frequency bands, and other participating regions within the same network.

### The importance of phase timing in neural transmission and cognitive functioning

As mentioned, our dissociating results here between the in-phase and anti-phase conditions are more in line with the direct-connection account^19^, which proposes that phase shifts are natural in neural communication due to the time it takes, as small as it may be, from region A to region B. Our rationale is that since the only difference between in-phase and anti-phase stimulation is their shift in timing, where even the maximal 180° shift only translates to a difference of 12.5 ms (half cycle in the 40 Hz gamma range) in time between two electrodes, the left temporal and parietal cortices are oscillating in a temporally-precise manner down to the millisecond level. This has also been observed both for gamma coherence between the frontal eye field (FEF) and V4^27^ and theta coherence between hippocampus and the medial prefrontal cortex^66^,^67^. To take a step further, Fell and Axmacher^19^ suggested that a temporal phase difference of 0° essentially means that there is absolutely no time lag between the two regions, which implies that such synchronization may not represent mutual information transmission. Instead, it may be that the two regions were receiving a common input from a third source, or it may be a result of a delayed interaction between inhibitory interneurons and excitatory pyramidal cells. Therefore, it should be emphasized that anti-phase tACS should not always be viewed as a tool for neural interruption in every context. If coherence between different brain regions entails that a consistent phase relation (instead of random noise) is maintained among the areas^38^, then consistent phase difference such as the case in Experiment 1 does not necessarily imply noise, but phase coherence with a consistent phase relation of 180°, which may be advantageous or disadvantageous depending on different contexts.

Physiological support of such account comes from one important study by Gregoriou *et al*.^27^. These authors reported that, as monkeys performed a visual detection task, their FEF and occipital areas (e.g., V4) exhibited a phase difference of 152° that roughly corresponds to a 8~13 ms lag (i.e., high gamma range) between the neural activities of these two regions, which was interpreted as the axonal conductance time and synaptic delays between the two areas. Critically, FEF activities always preceded those of V4, thus suggesting FEF to be the “driver” of such phase relationship due to its functional specialization in visual attention. In humans, Baldauf and Desimone^68^ also found gamma synchronization between the inferior frontal junction (IFJ) and more specialized areas such as the fusiform face area or the parahippocampal place area, depending on which stimulus (face or place) the participants were attending to at the time. Similarly, the IFJ also preceded other regional activities by 20 ms (i.e., gamma cycle). Thus, the authors concluded that the IFJ may direct the flow of visual processing via coupled oscillations with other specialized areas, and suggested phase shift as a possible neuronal mechanism for affecting distant cells at a time of maximum depolarization, which increases impact. To contextualize these findings by Desimone and colleagues^27^,^68^ with the present study, we interpret the left parietal cortex as the “driver” of the synchrony due to its specialized role in attention and working memory. This would be consistent with how Parra *et al*.^11^ interpreted their fMRI results: they suggested that fusiform gyrus, as a part of the temporal cortex that specializes in representing features, was working in concert with the parietal cortex, which served as “the glue” that kept features bound together in VWM. If these are correct, then together with Desimone and colleagues’ findings that have consistently observed an early phase shift in the attentional “driver” area, in the context of binding VWM the parietal cortex is likely to be the driver with early phase shift whereas specialized areas such as the fusiform gyrus would be a few ms (i.e., gamma cycle) behind. This will either require EEG or a tACS setup that is time-locked to the visual stimuli while being able to record its own current output. In the context of the present study, it is important to note that although here we report an enhancement effect with an anti-phase gamma setup, it is possible that the real optimal phase lag might be somewhere between 0° and 180°. Therefore, perhaps an even greater improvement effect in binding VWM performance can be observed if the optimal phase difference is tweaked accurately. Nevertheless, the current study at least demonstrates that 180° is already effective in improving low-performers’ binding VWM.

Beyond demonstrating the presence of interregional communication between the left temporal and parietal cortex in gamma frequency that is temporally-precise and binding-specific, results from the present study also highlight the possibility of *intra*-regional coherence in supporting cognitive functioning. That is, phase coherence *within* each individual brain area is likely to be an important factor that determines the outcome of the tACS effect as well. This speculation is made by comparing our tACS setup and findings with those by Jausovec and colleagues^31^,^32^,^39^. Jausovec and colleagues, with a consistent tACS setup of one electrode over either the left frontal or parietal cortex and the reference electrode over the right eyebrow, have reported improved visuospatial memory and fluid intelligence. From a strictly interregional perspective, these positive findings are hard to explain because synchronizing a brain region with the right frontal sinus and orbital cavity (or vice versa) is unlikely to generate any cognitive effect. Therefore, we speculate in such cases tACS effects are driven by the synchronization of neurons within each specific region (the frontal or parietal electrode) but not interregional. If true, then the effects of the present study, as well as many others, are likely driven by a combination of intra- and interregional coherence. It would be of great interest if future studies can systematically manipulate tACS electrode placements to map out the independent contribution of intra- and interregional synchronization.

### Brain stimulation and individual differences

The present study supports the view that preexisting individual differences should be taken into account when assessing the effect of brain stimulation. This is consistent with our previous work using anodal tDCS, where low-performers were able to improve their VWM performance after right parietal tDCS application^7^,^8^. In studies that have multiple levels of task difficulty, this effect can also be shown in conditions with higher task difficulty via the means of information complexity^69^, retrieval difficulty^31^, or attentional and motor interference^70^. Using tDCS, Wu *et al*.^70^ found that anodal tDCS was only facilitative to VWM performance in the condition where participants had to retrieve visual information in a backward sequence, with attentional and motor interference during the retention period. Combining the same task with tACS, Jausovec, Jausovec and Pahor^31^ also reported that theta tACS over left prefrontal cortex improved backward recall, which is consistent with the Wu *et al*. study. Santarnecchi and colleagues^69^ also found that gamma tACS over the prefrontal cortex and vertex was able to improve performance on Raven’s matrices task, but only in complex trials where conditional and logical reasoning was required. Together, these findings demonstrate that the effect of tDCS and tACS can interact with a variety of factors, including participants’ baseline performance, task difficulty, and the stimulated brain region.

However, does this mean that everyone, including our high-performers, can also improve their VWM performance when adequately challenged? Here it is important to note that the high-performers are only relatively better in terms of VWM performance, but objectively they have never hit the task ceiling either in the current or our previous studies^7^,^8^. In fact, their performance is far from perfect. Therefore, there seems to be a cognitive ceiling that tDCS or tACS, at least with the current protocol, is unable to push people beyond. This was indeed what we found electrophysiologically, where high-performers have reached an asymptote in EEG amplitude at the same time as their behavioral performance asymptotes^7^,^8^. Thus, to help the high-performers improve their performance, the answer may lie in optimized tACS protocol such as tACS of other frequency ranges^71^ instead of the cognitive task itself.

The dissociation between the effects of tACS in low- and high-performers also resembles something akin to the effects of stochastic resonance, which posits that the addition of low-level noise can sometimes improve signal-to-noise ratio because the noise supposedly pushes the subthreshold signals over the edge^72^,^73^,^74^. Indeed, one TMS study by Schwarzkopf *et al*.^75^ found that only low-intensity and not high-intensity TMS could improve performance in low-signal conditions, which can be viewed as equivalent to high task difficulty^70^ or low-performers^7^,^8^, both of which have been shown to benefit from tDCS. In the context of the present experiment, perhaps anti-phase coherence was closer to the optimal temporal difference within the temporoparietal network, and thus provided the optimally-low level of noise. This would also be consistent with our previous point that maybe the high-performers simply needed a stimulation protocol that was closer to their optimal low-noise level to improve their performance, all of which requires further investigation.

Clinically, the current finding in low-performers seems to be a promising start for application of tACS in clinical populations that are known to suffer from poor VWM. One unique case of specific impairment in feature-binding, but not single-feature, VWM is the Alzheimer’s disease^29^,^76^. In this special case, binding VWM task can even serve as an early screening task for people who are at risk for early-onset Alzheimer’s disease, with better predictive power than other standardized neurological tasks^76^. Therefore, it would be useful for future studies to apply the same tACS protocols to different patient populations that also suffer from poor VWM to investigate whether healthy low-performers are suitable models for designing clinical tACS parameters.

## Additional Information

**How to cite this article**: Tseng, P. *et al*. The critical role of phase difference in gamma oscillation within the temporoparietal network for binding visual working memory. *Sci. Rep*. **6**, 32138; doi: 10.1038/srep32138 (2016).
